# Targeting SERPINB5 and MSLN for Pancreatic Cancer Therapy

**DOI:** 10.3390/cancers18193242

**Published:** 2026-10-08

**Authors:** Jinghui Sun, Lingling Liu, Rhyston Broadhurst, David Wang, Anand Mehta, Denis C. Guttridge, Leonardo M. R. Ferreira, Haizhen Wang, Xueliang Gao

**Affiliations:** 1 Department of Pharmacology & Immunology, Medical University of South Carolina, Charleston, SC 29425, USA; sunji@musc.edu (J.S.); liulin@musc.edu (L.L.); broadhrh@musc.edu (R.B.); mehtaa@musc.edu (A.M.); ferreirl@musc.edu (L.M.R.F.); 2 Hollings Cancer Center, Medical University of South Carolina, Charleston, SC 29425, USA; wangdav@musc.edu (D.W.); guttridg@musc.edu (D.C.G.)

**Keywords:** pancreatic cancer, metastasis, SERPINB5, MSLN, combination treatment

## Abstract

**Background/Objectives**: Pancreatic cancer (PC) is a leading cause of cancer-related death in the United States and lacks efficient therapeutic options. Serine peptidase inhibitor, clade B, member 5 (SERPINB5) is non-inhibitory toward serine proteases, yet it has diverse biological functions, including regulating cell migration, cell death, gene expression, and stress responses. Mesothelin (MSLN) is a tumor-associated antigen that is broadly overexpressed on various malignant tumor cells and plays a crucial role in promoting proliferation, invasion, and metastasis. **Methods**: We integrated public database analyses, cell-line screening, gene knockdown and overexpression, immunoblotting, cell viability and apoptosis assays, flow cytometry, chromatin immunoprecipitation paired with quantitative PCR (ChIP-qPCR), and chimeric antigen receptor (CAR) T-cell therapy to investigate SERPINB5 and MSLN functions, developing a novel and effective therapeutic strategy for patients with pancreatic cancer. **Results**: Both SERPINB5 and MSLN are highly expressed and negatively associated with the overall survival of PC patients. Our mechanistic study suggests that SERPINB5 negatively regulates the mitochondrial protein dehydrogenase/reductase 2 (DHRS2) to promote PC cell survival; consequently, SERPINB5 depletion induces PC cell apoptosis. Of note, SERPINB5 depletion increases MSLN transcription by enhancing c-Myc binding to the MSLN promoter region, which in turn suppresses the mitochondrial protein DHRS2 and leads to resistance to apoptosis in PC cells. Our genetic ablation study shows that SERPINB5 and MSLN are critical for PC cell survival, and that depleting both proteins synergistically induces PC cell apoptosis. Gemcitabine (GEM) is a standard chemotherapy drug for PC treatment. Our study shows that SERPINB5-high PC cells display greater sensitivity to GEM than SERPINB5-low PC cells. GEM treatment increases mitochondrial DHRS2 protein expression, similar to the effect of SERPINB5 knockdown, whereas upregulated MSLN confers apoptotic resistance by decreasing DHRS2 levels. **Conclusions**: Our in vitro study demonstrates that sequential treatment with GEM followed by anti-MSLN chimeric antigen receptor (CAR)-T cells is an effective and promising strategy for patients with pancreatic cancer. Future studies using mouse models may validate the efficacy of this sequential treatment strategy in vivo.

## Simple Summary

Pancreatic cancer (PC) is a leading cause of cancer-related death, with no effective therapy for curing late-stage PC. Developing effective therapeutic strategies is an emergent unmet need. SERPINB5 is non-inhibitory toward serine proteases, and mesothelin (MSLN) is a tumor-associated antigen. SERPINB5 and MSLN are highly expressed and negatively associated with the overall survival of PC patients. This study shows that SERPINB5 depletion upregulates the mitochondrial protein DHRS2, inducing apoptosis in PC cells. Meanwhile, SERPINB5 depletion increases MSLN transcription, which in turn suppresses DHRS2 and leads to apoptosis resistance. Thus, depleting SERPINB5 and MSLN synergistically induces apoptosis in PC cells. This study shows that SERPINB5-high PC cells are more sensitive to gemcitabine. An in vitro study shows that sequential treatment with gemcitabine followed by anti-MSLN chimeric antigen receptor (CAR)-T cells effectively suppressed PC cell viability, supporting the idea that this combination is a promising therapeutic strategy for PC patients.

## 1. Introduction

Pancreatic cancer (PC) is an aggressive malignancy with a 5-year survival rate of approximately only 11% [1,2]. At diagnosis, more than 80% of PC patients have already lost the chance of surgery due to peripheral spread or remote metastasis. PC treatment involves chemotherapy, radiation therapy, vaccination, immunotherapy, and dietary changes, yet outcomes remain poor [3,4]. Developing efficient therapeutic strategies for PC patients is urgently needed [5].

The SERPIN gene encodes serine protease inhibitors, the largest family of protease inhibitors [6]. Unlike other SERPIN family members, SERPINB5 gene-encoded serpinB5 is non-inhibitory to serine proteases, but it has diverse biological functions in regulating cell migration, death, gene expression, and stress responses [7,8,9]. SERPINB5 plays distinct roles in different cancer types. Some clinical studies have shown a positive correlation between SERPINB5 expression and tumor progression in pancreatic, lung, ovarian, and breast cancers [10,11,12,13,14], whereas others have observed the opposite trend in prostate, breast, and lung cancers [15,16,17,18]. SERPINB5 is upregulated during pancreatic cancer progression, promoting its metastasis [10,19]. The MSLN gene encodes mesothelin, a tumor-associated cell surface antigen that is broadly overexpressed on malignant tumor cells and is an attractive candidate for targeted therapy. Aberrant MSLN expression on tumor cells plays an important role in promoting proliferation, invasion, and metastasis [20,21,22]. MSLN is highly expressed in pancreatic cancer tissues, but not in para-cancerous tissues [23]. MSLN-expressing tumors have been reported to be associated with increased microvascular density in pancreatic metastatic tissues [24]. The level of serum MSLN has been considered a potential diagnostic and prognostic biomarker for pancreatic cancer [25].

SERPINB5 and MSLN are highly expressed and negatively associated with the survival of patients with high-stage PC. Our study shows that depletion of SERPINB5 or MSLN induces PC cell apoptosis. Thus, simultaneously targeting SERPINB5 and MSLN is a promising avenue in treating PCs. Currently, no inhibitor or compound specifically targets SERPINB5. Our study shows that high-SERPINB5 PC cell lines exhibit increased apoptosis and reduced viability upon gemcitabine (GEM) treatment. Meanwhile, dehydrogenase/reductase SDR family member 2 (DHRS2) lowers mitochondrial reactive oxygen species and alters the NADP/NADPH ratio to control cellular stress. Upregulation of DHRS2 not only manages cellular stress but also induces cancer cell death and growth inhibition by stabilizing the tumor suppressor p53 or suppressing metabolic oncogenes such as Choline Kinase α (CHKα) and/or Sphingosine Kinase 1 (SPHK1) [26,27,28,29]. DHRS2 overexpression inhibits renal cancer cell proliferation by inducing a severe loss of mitochondrial membrane potential [30]. Mitochondrial dysfunction increases cancer cell apoptosis and/or blocks cancer progression [31,32]. Therapeutic agents such as histone deacetylase inhibitors or SUV420H2 inhibitors upregulate DHRS2 expression, overcoming drug resistance and sensitizing cancer cells to apoptosis [33]. These findings suggest that DHRS2 upregulation may drive apoptosis in PC cells. Our study suggests that SERPINB5 knockdown or GEM treatment enhances DHRS2 protein expression, thereby inducing PC apoptosis. Meanwhile, upregulated MSLN downregulates DHRS2 expression, leading to apoptosis resistance.

Strikingly, our study shows that sequential treatment with GEM and anti-MSLN CAR-T cells significantly decreases PC cell survival in vitro. Thus, combining GEM with anti-MSLN CAR-T cells is promising for treating PC.

## 2. Materials and Methods

### 2.1. Antibodies

The following antibodies were used in this study. SERPINB5 (Proteintech (Rosemont, IL, USA), 11722-1-A, RRID: AB_10597111), MSLN (mesothelin) (Proteintech, 66404-1-lg, RRID: AB_2881778), BaX (Proteintech, 50599-2-Ig, RRID: AB_2061561), cleaved caspase-3 (Cell Signaling Technology (Danvers, MA, USA), 9664, RRID:AB_2070042), cleaved PARP (Cell Signaling Technology, 9541, RRID:AB_331426), Phospho-ATR (Thr1989) (Cell Signaling Technology, 30632T, RRID: AB_2798992), Phospho-Chk1 (Ser345) (Cell Signaling Technology, 2348T, RRID: AB_331212), Chk1 (Cell Signaling Technology, 2360T, RRID: AB_2080320), DHRS2 (Proteintech, 15735-1-AP, RRID: AB_2091847), c-Myc (Thermo Fisher (Waltham, MA, USA), 9E10, MA1-980), beta-actin (Proteintech, 66009-1-lg, RRID: AB_2687938), FLAG (Sigma-Aldrich (St. Louis, MO, USA), F1804, RRID: AB_262044), APC-annexin V (Thermo Fisher, 88-8007-74, RRID: AB_2575166).

### 2.2. Cells

HEK293T cells (RRID: CVCL_0063), BxPC-3 (RRID: CVCL_0186), CFPAC-1 (RRID: CVCL_1119), PANC-1 (RRID: CVCL_0480), and MIA PaCa-2 (RRID: CVCL_0428) cell lines were purchased from the American Type Culture Collection (ATCC) (Manassas, VA, USA). Hs766T (RRID: CVCL_0334), AsPC-1 (RRID: CVCL_0152), and Capan-1 (RRID: CVCL_0237) were purchased from the Tissue Culture Facility (TCF) at MUSC. These cells have been authenticated to NIH standards and are free of mycoplasma. BxPC-3, MIA PaCa-2, PANC-1, Hs766T cells were cultured in Dulbecco’s Modified Eagle Medium (DMEM), CFPAC-1 and Capan-1 cells were cultured in Iscove’s Modified Dulbecco’s Medium (IMDM, Cytiva (Marlborough, MA, USA)), and AsPC-1 cells were cultured in Roswell Park Memorial Institute Medium 1640 (RPMI-1640; Hyclone). All culture media were supplemented with 10% fetal bovine serum (FBS) (Thermo Fisher), except for Capan-1 cells, which were supplemented with 20% FBS, and 1% penicillin/streptomycin. These cells were maintained under standard culture conditions at 37 °C in humidified air with 5% CO_2_. None of these lines are listed in the Database of Cross-Contaminated or Misidentified Cell Lines.

### 2.3. Quantitative PCR

Total RNA was extracted using an RNA RNeasy mini kit (Qiagen (Venlo, The Netherlands)). Subsequently, 500 ng of RNA was reverse transcribed into cDNA using reverse transcriptase (Bio-Rad (Hercules, CA, USA)). Quantitative reverse-transcription polymerase chain reaction (qRT-PCR) was performed using the CFX96 Touch Real-Time PCR Detection System (Bio-Rad) with specific primers. The expression levels were normalized against GAPDH and analyzed using the 2^−ΔΔCt^ method to calculate fold changes in gene expression. Eurofins Genomics LLC synthesized primers with the following sequences: MSLN, forward 5′-CCAACCCACCTAACATTTCCAG-3′, reverse 5′-CAGCAGGTCCAATGGGAGG-3′; and GAPDH, forward 5′-TGTGGGCATCAATGGATTTGG-3′, reverse 5′-ACACCATGTATTCCGGGTCAAT-3′.

### 2.4. Chromatin Immunoprecipitation (ChIP)-qPCR

The ChIP experiments were performed using ChIP-IT^®^ Express Enzymatic Kit (Active Motif (Carlsbad, CA, USA)). The precipitation of MSLN promoters was determined by PCR using primers spanning the regions indicated in the figure. The primer pairs for the PCR reaction in the ChIP with the MSLN promoter were listed below. ChIP-qPCR enrichment was expressed as percent input, and an IgG control was included as a background control. Three independent biological experiments were performed.

Primer 1—forward: 5′-AGAGTGGGATCCCGTGAAAC-3′

Primer 1—reverse: 5′-GCGCTGAGCCTCCTCTG-3′

Primer 2—forward: 5′-CTCCACCCACACATTCCTGG-3′

Primer 2—reverse: 5′-GCGCTGAGCCTCCTCTG-3′

Primer 3—forward: 5′-CTGTCCAAGGCTCCTGTGC-3′

Primer 3—reverse: 5′-GGGCCGTCAAAGGGAACAAA-3′

Primer 4—forward: 5′-CTCAGCGCCACGCACT-3′

Primer 4—reverse: 5′-TGTGTAGATCCCAGGGAGGG-3′

### 2.5. Lentiviral Packaging and Infection

The shRNA-pLKO.1 expression plasmids (Sigma-Aldrich) were packaged in HEK293T cells. Two individual shRNA were applied for the knockdown of SERPINB5 (shSERPINB5-#1: TRCN0000373444 and shSERPINB5-#2: TRCN0000373505) or MSLN (shMSLN-#1: TRCN0000245472 and shMSLN-#2: TRCN0000245474). Lipofectamine 2000 (Thermo Fisher) was used for the transfection of HEK293T cells. pCMV-VSVG: pCMV-delta8.9: expression vector was mixed at a ratio 1:5:10 and transfected in HEK293T cells. Pancreatic cancer cells were infected with 0.5 mL lentivirus-containing medium for 24 h in the presence of 10 μg/mL polybrene. Forty-eight hours later, puromycin (1 μg/mL) was added to the culture medium for selection. Plasmids for MSLN (OHu210460), SERPINB5 (OHu16606), and DHRS2 (OHu02326D) were ordered from GenScript (Piscataway, NJ, USA).

### 2.6. Immunoblotting

In brief, total cellular proteins were extracted from harvested cells using RIPA buffer supplemented with protease and phosphatase inhibitors. Protein concentrations were quantified with a BCA protein assay (Thermo Fisher) and measured with a BioTek Synergy H1 Hybrid Reader microplate spectrophotometer (Agilent, Winooski, VT, USA). Cellular protein was separated in 10–15% SDS-PAGE and transferred to 0.45 μm nitrocellulose membranes. Membranes were blocked with 5% BSA in TBS for 1 h before overnight incubation with primary antibodies at 4 °C. After three washes with TBS-0.1% Tween-20, membranes were incubated with secondary antibodies at room temperature for 1 h. Chemiluminescent signals were detected using a CCD camera-based imaging system (Odyssey, LI-COR Biosciences (Lincoln, NE, USA)) and analyzed with Image Studio software (LI-COR, version 5.2.5). The presented immunoblot data are representative of at least three independent experiments. The densitometry values were obtained using ImageJ v1.54p. Equal-sized ROIs were applied to each band, and a nearby blank region was used for background correction. The corrected intensities were expressed relative to the corresponding control lane, which was set to 100.

### 2.7. Flow Cytometry

BxPC-3, CFPAC-1 PANC-1, AsPC-1, and MIA PaCa-2 cells were seeded in 6-well plates (2 × 10^5^ cells/well). Cells were treated with drugs for 72 h. Both adherent and floating cells were harvested and washed twice with cold PBS. Cells were then resuspended in 1× Annexin V binding buffer (BD Biosciences, (San Jose, CA, USA)) at a concentration of 1 × 10^6^ cells/mL. Following this, 100 µL of cell suspension was transferred to a 5 mL culture tube, and 5 µL of FITC Annexin V and 5 µL of PI solution (BD Biosciences) were added. The mixture was gently vortexed and incubated for 15 min at room temperature in the dark. Following incubation, 400 µL of 1× binding buffer was added. Flow cytometric analysis was immediately performed using a BD FACSCanto II flow cytometer (BD Biosciences), and data were collected from at least 10,000 events per sample. Cells were gated based on forward and side scatter properties to exclude debris. The percentages of viable (Annexin V^−^/PI^−^), early apoptotic (Annexin V^+^/PI^−^), late apoptotic (Annexin V^+^/PI^+^), and necrotic (Annexin V^−^/PI^+^) cells were quantified. Data analysis was performed using FlowJo software (version 10.0.7, Tree Star Inc. (Ashland, OR, USA)), and all experiments were conducted in triplicate.

### 2.8. Cytotoxicity Assay

Cells were seeded in 96-well plates and allowed to attach overnight. The following day, cells were treated with gemcitabine at the indicated concentrations for 72 h. Cell viability was assessed using the Cell Counting Kit-8 (CCK-8) assay according to the manufacturer’s instructions, and absorbance was measured at 450 nm. For each independent experiment, blank absorbance was subtracted and cell viability was normalized to the untreated control (100%). Technical replicates were averaged, and normalized data from three independent experiments were jointly fitted using a four-parameter logistic model with variable slope in GraphPad Prism version 10.0.3. The Bottom was constrained to values greater than 0, while the Top and Hill slope were unconstrained. IC_50_ was defined as the concentration corresponding to the midpoint between the fitted Top and Bottom of the dose–response curve. Data are presented as mean ± SD unless otherwise specified.

### 2.9. Anti-MSLN CAR T-Cell Preparation and Killing Assay

The anti-MSLN-CAR-2A-GFP lentiviral plasmid was synthesized by VectorBuilder (Chicago, IL, USA). The EF1A promoter was used. The anti-MSLN CAR gene contained a CD8a signal peptide, the anti-MSLN SS1 single-chain variable fragment (scFv) sequence obtained from US patent US20150031624A1 [34], a CD8 hinge and transmembrane domain, and a 41BB-CD3z signaling domain. The sequence is available upon request. Lentivirus was produced by transfecting HEK293FT cells with the CAR plasmid and lentiviral packaging plasmids, as previously described [35]. Lentivirus was titrated in Jurkat cells, aliquoted, and stored at −80 °C until use [35]. Human CD4+ and CD8+ T cells were isolated from peripheral blood mononuclear cells (PBMCs) obtained from de-identified blood donors (STEMCELL Technologies, Vancouver, BC, Canada) by magnetic negative selection using Human CD4+ T Cell Isolation Kit and Human CD8+ T Cell Isolation Kit (STEMCELL Technologies), respectively, following manufacturer’s instructions. Isolated CD4+ and CD8+ T cells were activated with anti-CD3/CD28 dynabeads (Gibco) at a 1:1 bead to cell ratio and 100 IU/mL IL-2 for CD4+ T cells and 300 IU/mL IL-2 for CD8+ T cells. Two days after activation, CD4+ and CD8+ T cells were transduced separately with anti-MSLN CAR lentivirus at a multiplicity of infection (MOI) of 1 (one particle per cell) in the presence of 100 IU/mL IL-2 or 300 IU/mL IL-2, respectively, and centrifuged at 1000× *g* at 32 °C for 1 h, as previously described [35]. Following transduction, CD4+ and CD8+ T cells were maintained and expanded in RPMI-10 medium with fresh medium and IL-2 being given every 2 days. Transduction efficiency was evaluated using flow cytometry to detect reporter GFP expression.

For in vitro cytotoxicity assays, 1 × 10^5^ cancer cells per well were seeded in 24-well plates. On day 2, cells were treated with gemcitabine for 24 h. Anti-MSLN CAR T-cells were pre-cultured in RPMI-1640 medium with 300U hIL2. On day 3, the gemcitabine-containing medium was replaced with fresh medium. Anti-MSLN CAR CD4+ and CD8+ T cells were combined at a 1:1 ratio, normalized for the frequency of CAR+ cells, and added to target cancer cells at effector to target (E:T) ratio of 1:4 for another 24 h. Untransduced CD4+ and CD8+ T cells were used as negative controls. Cell death was analyzed using the Annexin V/PI staining method.

### 2.10. Statistical Analyses

Results were presented as mean ± SD. Data analyses were performed with the two-tailed *p*-value or one-way ANOVA, or two-way ANOVA with correction for multiple comparisons, as appropriate. If not specified above, *p* < 0.05 was considered statistically significant. * *p* < 0.05, ** *p* < 0.01, *** *p* < 0.001, **** *p* < 0.0001 and n.s., not significant.

## 3. Results

### 3.1. SERPINB5 and MSLN Are Highly Expressed in PC Tissues and Are Negatively Associated with the Survival of High-Stage PC Patients

**Figure 1:**
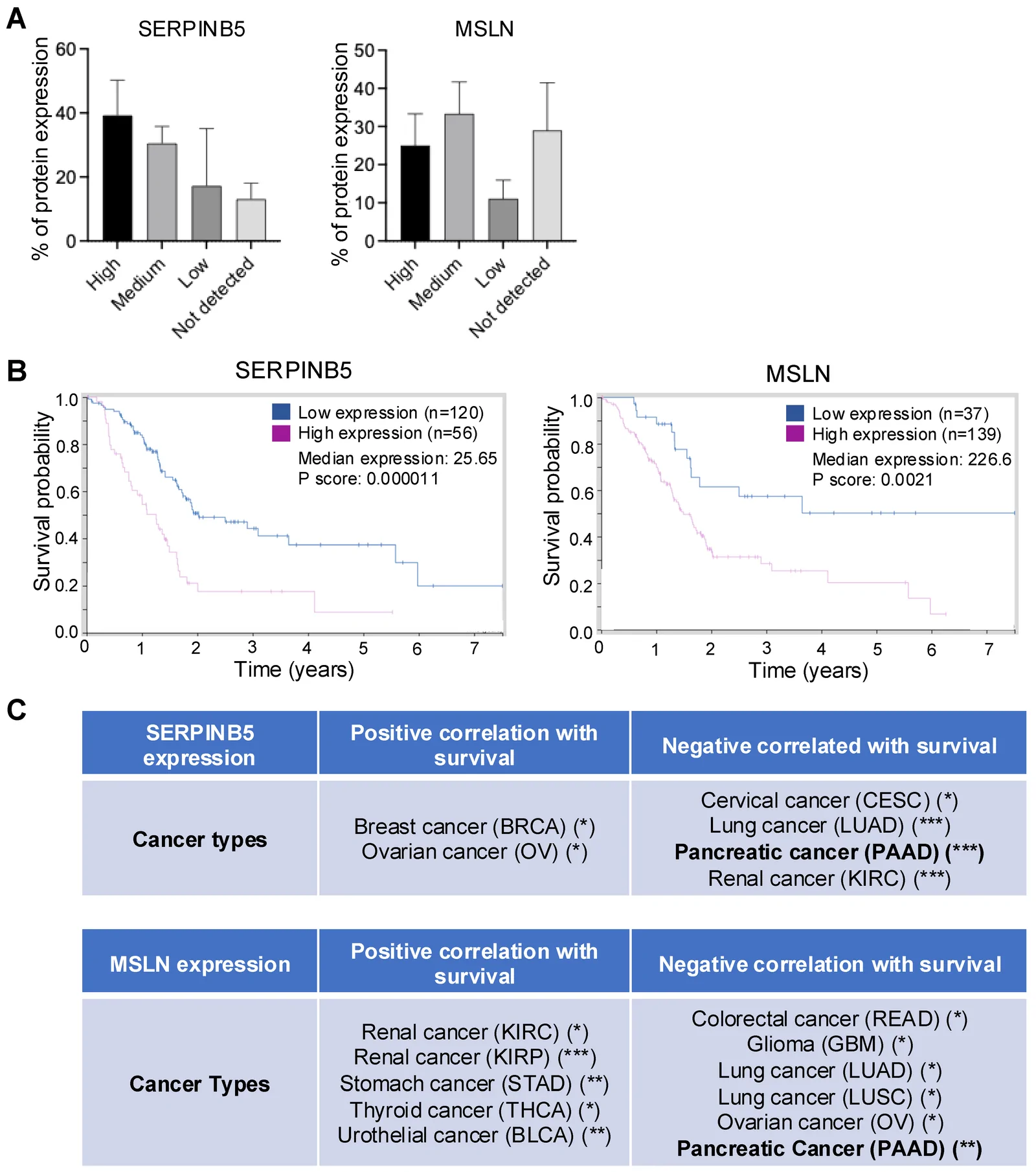
Data from the Human Protein Atlas show that SERPINB5 and MSLN are negatively associated with survival in patients with pancreatic cancer. (**A**) Protein expression levels were determined by immunohistochemistry (IHC) on PC patient samples using specific antibodies. Over 50% of pancreatic cancers express high/medium levels of SERPINB5 and MSLN. For SERPINB5, four different antibodies were applied for IHC. For MSLN, three different antibodies were applied for IHC. (**B**) SERPINB5 and MSLN expression are significantly negatively associated with the survival probability in patients with pancreatic cancer. (**C**) Correlation analysis between cancer patient survival probability and SERPINB5 or MSLN expression. In panel (**C**), bold text highlights pancreatic cancer, the focus of this study. * *p* < 0.05, ** *p* < 0.01, *** *p* < 0.001.

Immunohistochemistry (IHC) data from the Human Protein Atlas (proteinatlas.org) showed that PC samples frequently exhibited high expression of SERPINB5 (39.2%, *n* = 11) or MSLN (25.0%, *n* = 12), respectively (Figure 1A). Both SERPINB5 and MSLN were significantly and negatively correlated with the overall survival of PC patients (Figure 1B). Similar negative correlations between SERPINB5/MSLN expression and overall survival were also observed in patients with lung adenocarcinoma (LUAD), but not in other cancer types studied (Figure 1C). These findings suggest that targeting SERPINB5 and/or MSLN may be a promising therapeutic strategy for PC patients. 

### 3.2. SERPINB5 Suppresses MSLN Expression in PC Cells

**Figure 2:**
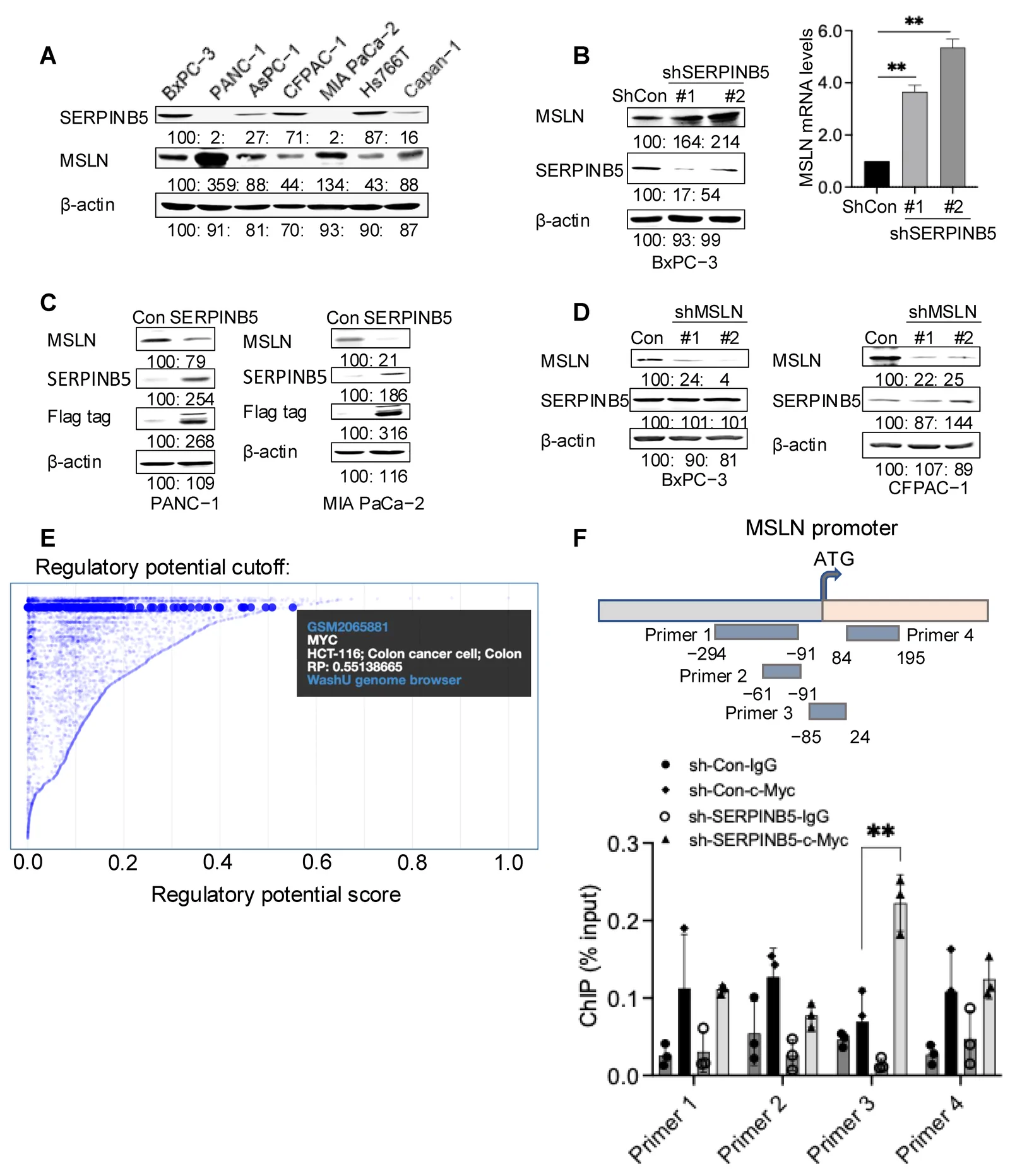
SERPINB5 suppresses MSLN expression, but not vice versa. (**A**) Immunoblotting using specific antibodies to examine the intrinsic expression of SERPINB5 and MSLN in seven pancreatic cancer cell lines. β-actin was a loading control. (**B**) SERPINB5 knockdown using shRNA (#1 and #2) significantly increased MSLN protein levels in BxPC-3 cells measured by immunoblot. (**C**) Immunoblot analysis showed that overexpressing SERPINB5 decreased MSLN expression in both PANC-1 and MIA PaCa-2 cell lines. (**D**) Immunoblots showed that MSLN knockdown using two individual shRNAs (#1 and #2) did not affect SERPINB5 expression in either BxPC-3 or CFPAC-1 cells. (**E**) The Cistrome Data Browser Toolkit (http://cistrome.org/db/#/) (accessed on 1 August 2024) was applied to discover transcription factors that regulate MSLN. Regulatory potential is a score reflecting the likelihood that Myc regulates MSLN. (**F**) **Upper**: Primers designed to span the MSLN promoter regions were indicated. **Lower**: c-Myc binding site to the MSLN promoter region was determined using a ChIP assay followed by RT-PCR. ** *p* < 0.01.

We analyzed SERPINB5 and MSLN protein expression levels in seven pancreatic cancer cell lines. BxPC-3, CFPAC-1, and Hs766T cells exhibited high SERPINB5 expression with medium or low MSLN expression (Figure 2A and Figure S1A). In contrast, PANC-1 and MIA PaCa-2 cells showed very low or undetectable SERPINB5 expression, and AsPC-1 and Capan-1 cells displayed low SERPINB5 expression; however, all four cell lines exhibited high or medium MSLN expression levels (Figure 2A). These findings indicate that SERPINB5 expression is negatively correlated with MSLN expression in most PC cell lines examined.

To test whether downregulating SERPINB5 increases MSLN expression, we examined MSLN levels in SERPINB5-knockdown BxPC-3 cells. MSLN expression was upregulated in SERPINB5-knockdown cells (Figure 2B and Figure S1B). Conversely, overexpression of SERPINB5 decreased MSLN expression in PANC-1 and MIA PaCa-2 cells (Figure 2C and Figure S1C). Notably, immunoblot analysis showed that MSLN knockdown did not affect SERPINB5 expression in BxPC-3 and CFPAC-1 cells (Figure 2D and Figure S1D). These results suggest that SERPINB5 negatively regulates MSLN expression in PC cells, but not vice versa.

c-Myc is a potent transcription factor that regulates the expression of at least 15% of the entire genome [36,37,38]. Data retrieved from http://cistrome.org/db/#/ (accessed on 1 August 2024) indicated that c-Myc has strong potential to bind the MSLN promoter region (Figure 2E). We therefore designed four primer pairs targeting different regions of the MSLN promoter for a ChIP assay to determine whether c-Myc binds to the MSLN promoter. The ChIP assay showed that c-Myc significantly increased its interaction with the −85 to 24 region of the MSLN promoter when SERPINB5 was knocked down (Figure 2F). These findings suggest that SERPINB5 inhibits MSLN transcription by interfering with c-Myc binding to the MSLN promoter region.

### 3.3. SERPINB5 and MSLN Are Critical for PC Cell Survival

**Figure 3:**
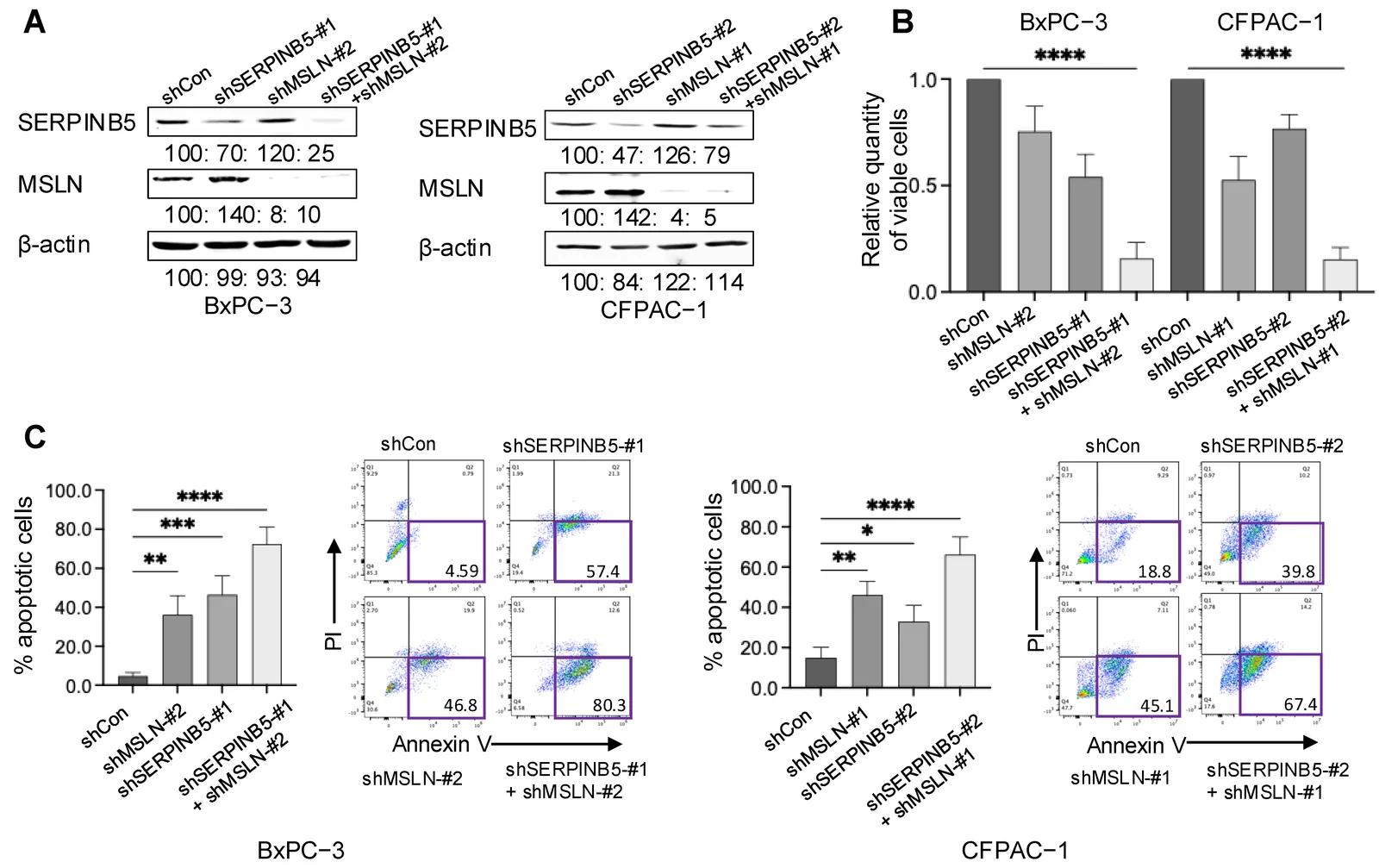
SERPINB5 and MSLN are critical for PC cell survival. (**A**) Immunoblot analysis of simultaneous knockdown of SERPINB5 and MSLN in BxPC-3 and CFPAC-1. Two sets of shRNA for SERPINB5 (shSERPINB5-#1 and -#2) and MSLN (shMSLN-#1 and -#2) were applied. (**B**) Cell viability was measured with CCK-8 assays in SERPINB5 and/or MSLN knockdown BxPC-3 and CFPAC-1 cells. (**C**) Flow cytometry analysis of apoptosis in SERPINB5 and/or MSLN-depleting BxPC-3 and CFPAC-1 cells. Apoptosis was assessed using Annexin V-PI staining and analyzed by flow cytometry. Different bar shades represent the experimental groups indicated on the x-axis. * *p* < 0.05, ** *p* < 0.01, *** *p* < 0.001, **** *p* < 0.0001.

To investigate SERPINB5’s role in PC cells, we knocked down SERPINB5 using two non-overlapping shRNAs and assessed cell viability. BxPC-3 and CFPAC-1 cell lines were selected because they express both SERPINB5 and MSLN. SERPINB5 knockdown dramatically decreased BxPC-3 and CFPAC-1 cell viability compared with control cells (Figure 3A,B and Figure S2). We next examined MSLN function in these cell lines by knocking down MSLN with two specific shRNAs. The viability of MSLN knockdown cells was also substantially decreased. Importantly, simultaneous depletion of SERPINB5 and MSLN markedly reduced PC cell viability (Figure 3A,B and Figure S2). Consistently, Annexin V/PI staining demonstrated that knockdown of both SERPINB5 and MSLN induced significant apoptosis in both cell lines (Figure 3C).

### 3.4. High SERPINB5-Expressing PCs Are Sensitive to Gemcitabine Treatment, and High MSLN Expression Leads to Gemcitabine Resistance

**Figure 4:**
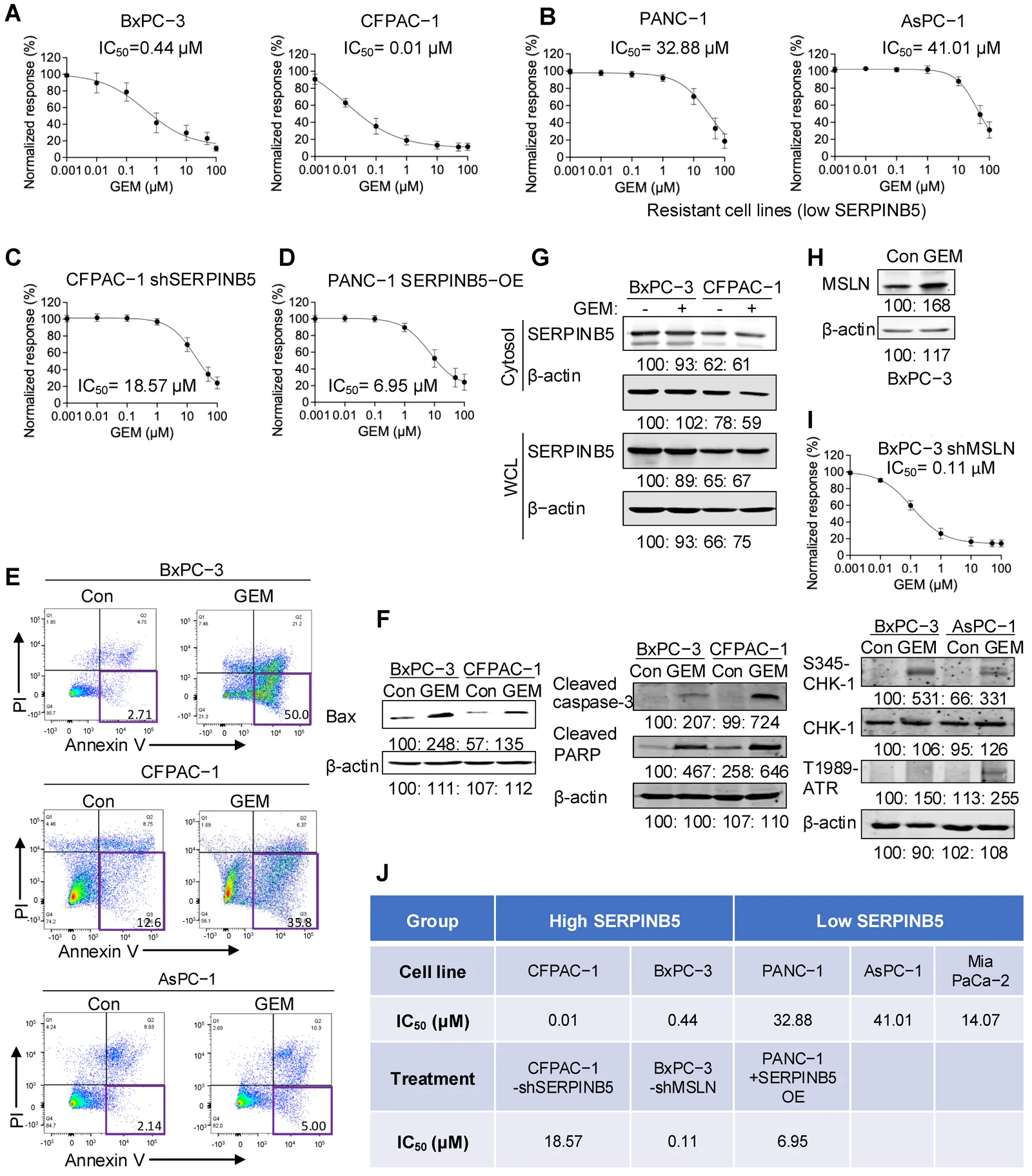
High SERPINB5-expressing PC cells are sensitive to gemcitabine treatment. (**A**,**B**) Cell viability was assessed using CCK-8 assays, which showed that high SERPINB5-expressing cell lines, BxPC-3 and CFPAC-1 (**A**), were more sensitive to escalating gemcitabine treatment (72 h) than low SERPINB5 cell lines, PANC-1 and AsPC-1 (**B**). (**C**,**D**) Cell viability of SERPINB5 knockdown CFPAC-1 cells (**C**) and SERPINB5 overexpressing-PANC-1 cells (**D**) upon gemcitabine treatment for 72 h. (**E**) Representative flow cytometry data were used to measure apoptosis in high intrinsic SERPINB5-expressing BxPC-3, CFPAC-1, and low SERPINB5-expressing AsPC-1 cells treated with 10 µM Gemcitabine for 72 h. Purple frames indicate apoptotic cell populations. (**F**) Immunoblotting showed that gemcitabine treatment (10 µM, 72 h) increased Bax, cleaved caspase-3, and PARP expression in high SERPINB5-expressing cells, BxPC-3, and CFPAC-1; and phosphorylation of S345-Chk1 and T1989-ATR in BxPC-3 and AsPC-1 cells. (**G**) Immunoblot of cytosolic or cellular amounts of SERPINB5 in BxPC-3 and CFPAC-1 cells receiving gemcitabine treatments (10 µM, 72 h). β-actin was the loading control. WCL: whole-cell lysate. (**H**) Immunoblot results showed increased MSLN expression in BxPC-3 cells after gemcitabine treatment (10 µM, 72 h). (**I**) Cell viability was measured with CCK-8 assays of MSLN knockdown BxPC-3 cells under gemcitabine treatment for 72 h. (**J**) The overall results showed that high intrinsic SERPINB5-expressing PC cells display a lower IC_50_ than low SERPINB5-expressing cells in response to gemcitabine treatment. Knockdown of SERPINB5 increased, and overexpression of SERPINB5 decreased the IC_50_ for gemcitabine. MSLN knockdown reduced the gemcitabine IC_50_.

No commercially available chemical compounds or inhibitors specifically target SERPINB5. Gemcitabine (GEM) is a synthetic pyrimidine nucleoside analog used to treat multiple cancers, including pancreatic, lung, breast, colon, and ovarian cancers, either as a single agent or in combination [39]. GEM is also a standard chemotherapy regimen for patients with pancreatic cancer. We therefore examined the viability of PC cells following treatment with escalating doses of gemcitabine. The IC_50_ of GEM treatment for high SERPINB5-expressing BxPC-3 and CFPAC-1 cells was dramatically lower than that of low SERPINB5-expressing PANC-1 and AsPC-1 cells (Figure 4A,B). Of note, SERPINB5 depletion in CFPAC-1 cells reduced their sensitivity to gemcitabine treatment, increasing the IC_50_ from 0.01 µM to 18.57 µM (Figure 4A,C). Conversely, overexpressing SERPINB5 in PANC-1 cells significantly increased their sensitivity to gemcitabine, decreasing the IC_50_ from 32.88 µM to 6.95 µM (Figure 4B,D). These results indicate that PC cell sensitivity to gemcitabine depends on SERPINB5 expression. 

We next examined whether and how GEM treatment induces PC cell apoptosis. GEM treatment induced apoptosis in approximately 50% of BxPC-3 and 35% of CFPAC-1 cells over 72 h; both cell lines display high SERPINB5 expression. In contrast, AsPC-1, which displays low SERPINB5 expression, showed apoptosis in only about 5% of cells (Figure 4E). Consistently, under GEM treatment, BxPC-3 and CFPAC-1 showed enhanced expression of Bax, cleaved caspase-3, and PARP, classic molecular markers of apoptosis (Figure 4F and  Figure S3A). GEM treatment also increased phosphorylated S345-Chk1 and/or phosphorylated T1989-ATR, key biomarkers of the DNA damage response, in BxPC-3 and AsPC-1 cells (Figure 4F). Cytoplasmic SERPINB5 expression has been reported to predict unfavorable prognoses in cancer patients [40]. We therefore asked whether GEM regulates SERPINB5 subcellular localization. No significant changes in the nuclear and cytosolic levels of SERPINB5 were observed in BxPC-3 and CFPAC-1 cells treated with GEM (Figure 4G and Figure S3B), suggesting that GEM does not induce PC cell apoptosis by altering SERPINB5 subcellular localization. Notably, GEM treatment significantly increased MSLN expression (Figure 4H and Figure S3C), similar to that observed in SERPINB5 KD cells (Figure 2B). Meanwhile, MSLN KD increased sensitivity to GEM in BxPC-3 cells, decreasing the IC_50_ from 0.44 µM to 0.11 µM (Figure 4I). Together with data showing that high MSLN PC cells exhibited a higher IC_50_ for GEM (Figure 2A and Figure 4J), these findings indicate that intrinsic or treatment-induced high MSLN expression may confer PC cell resistance to GEM.

### 3.5. SERPINB5 Knockdown or Gemcitabine Treatment Increases DHRS2 Expression, Thereby Promoting Apoptosis

**Figure 5:**
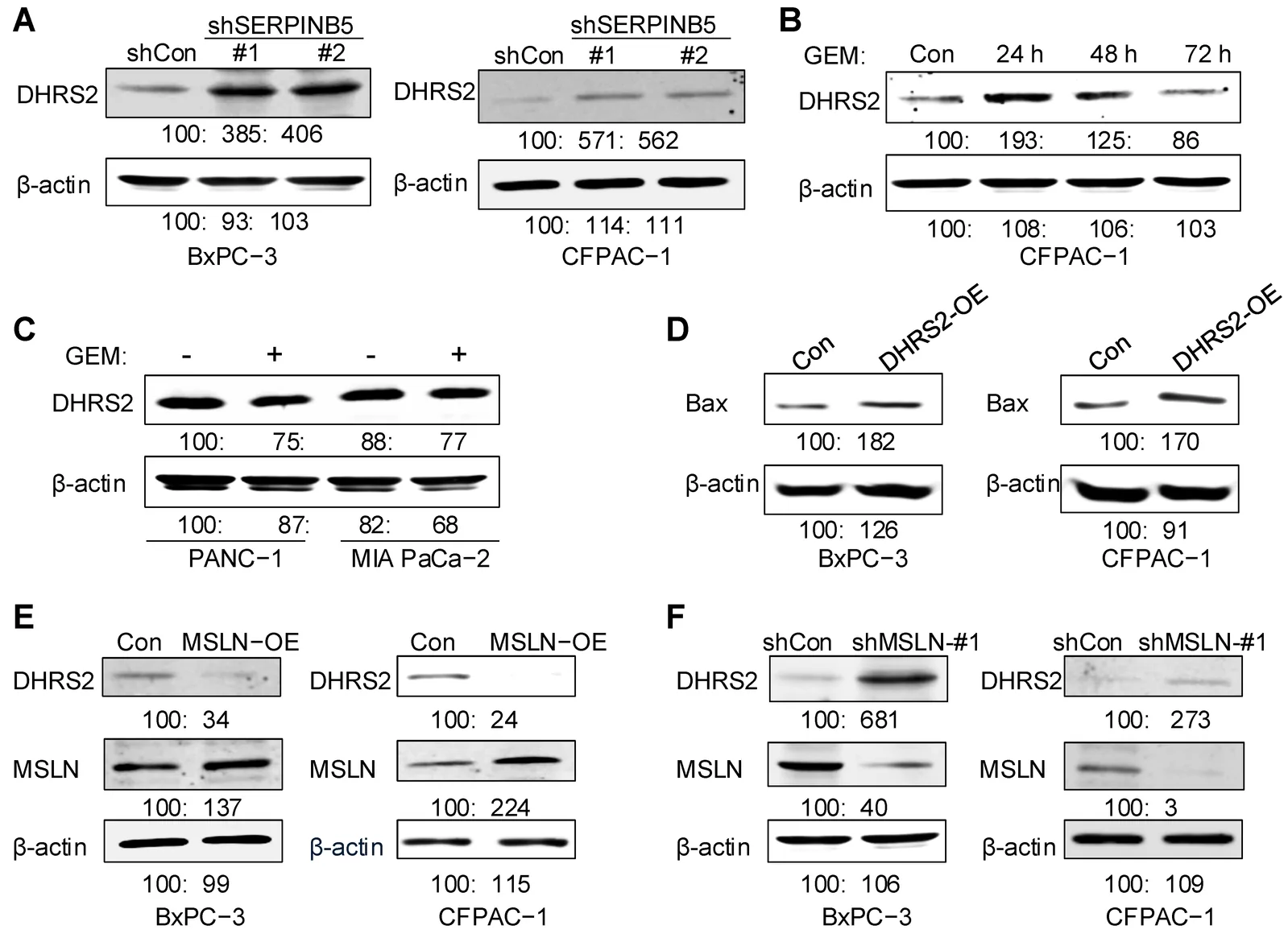
SERPINB5 knockdown increases the expression of the mitochondrial protein DHRS2, inducing PC cell apoptosis. (**A**) Immunoblot showed that SERPINB5 knockdown using shRNA significantly increased DHRS2 expression in BxPC-3 and CFPAC-1 cells. (**B**,**C**) Immunoblots showed that gemcitabine treatment (24 h) increased DHRS2 expression in CFPAC-1 cells (**B**) but not in PANC-1 or MIA PaCa-2 cells (**C**). (**D**) Immunoblots showed that overexpressing DHRS2 increased Bax expression in BxPC-3 and CFPAC-1 cells. (**E**,**F**) Immunoblots showed that MSLN overexpression decreased DHRS2 (**E**), whereas MSLN knockdown increased DHRS2 (**F**) in BxPC-3 and CFPAC-1 cells.

DHRS2 is an NADPH-dependent dicarbonyl reductase and can serve as a predictive biomarker for drug response. Increased DHRS2 expression inhibits cancer cell proliferation and promotes apoptosis [27,41,42]. Knockdown of SERPINB5 with two distinct shRNAs increased DHRS2 expression in BxPC-3 and CFPAC-1 cells (Figure 5A and Figure S4A). Consistently, GEM treatment increased DHRS2 expression in high-SERPINB5-expressing CFPAC-1 cells (Figure 5B and Figure S4B), whereas this effect was not observed in low-SERPINB5-expressing PANC-1 or MIA PaCa-2 cells (Figure 5C and Figure S4C). Meanwhile, GEM treatment increased Bax expression in high-SERPINB5-expressing cells but not in low-SERPINB5-expressing cells (Figure 4F). Consistently, transient overexpression of DHRS2 increased Bax expression (Figure 5D and Figure S4D). Given that SERPINB5 has been reported to regulate protein expression, we reasoned that GEM treatment or SERPINB5 knockdown in high-SERPINB5-expressing PC cells upregulates DHRS2 expression, thereby promoting cancer cell apoptosis.

SERPINB5 knockdown or GEM treatment increased MSLN expression (Figure 2B and Figure 4H). Simultaneous knockdown of both SERPINB5 and MSLN further increased cell apoptosis (Figure 3C). MSLN knockdown alone enhanced sensitivity to GEM treatment (Figure 4I,J). Notably, MSLN overexpression decreased DHRS2 protein expression, whereas MSLN knockdown increased DHRS2 expression (Figure 5E,F and Figure S4E,F). These results suggest that MSLN upregulation in GEM-treated cells downregulates DHRS2, thereby promoting apoptosis resistance.

### 3.6. Sequential Combination of Gemcitabine and Anti-MSLN CAR-T Cells Efficiently Suppresses Pancreatic Cancer Cell Viability

**Figure 6:**
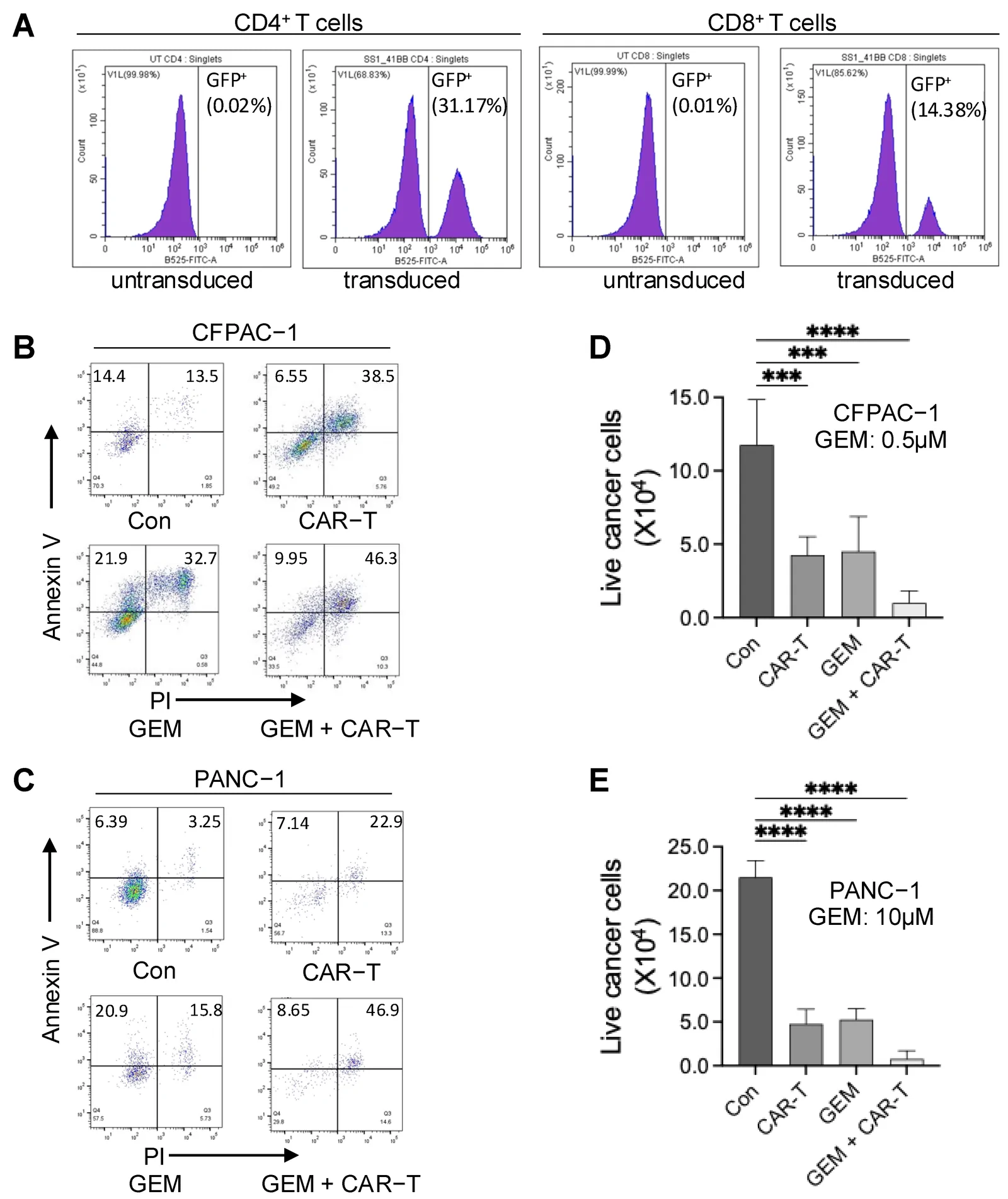
Sequential treatment of gemcitabine and anti-MSLN CAR-T cells efficiently suppresses pancreatic cancer cell survival. (**A**) Transduction efficiency of human CD4^+^ and CD8^+^ T cells with anti-MSLN CAR lentivirus, as determined by the frequency of reporter GFP^+^ cells. (**B**,**C**) Representative flow cytometry data showed that cell death was significantly increased when pancreatic cancer cells were sequentially treated with gemcitabine and anti-MSLN CAR T cells. A total of 0.5 µM of gemcitabine was applied for CFPAC1 (**B**), and 10 µM gemcitabine was applied for PANC-1 (**C**). Cell death was assessed using the APC-Annexin V/PI staining kit, followed by flow cytometry. (**D**,**E**) Surviving cells were analyzed after the same treatment as indicated in (**B**,**C**). *** *p* < 0.001, **** *p* < 0.0001.

High-SERPINB5-expressing pancreatic cancer cells showed high sensitivity to GEM treatment, inducing apoptosis. However, GEM treatment augments MSLN expression, thereby promoting GEM resistance (Figure 4 and Figure 5). Anti-MSLN CAR-T cells can eliminate MSLN-expressing cancer cells. We therefore tested whether combining GEM with anti-MSLN CAR T-cells could effectively suppress pancreatic cancer cell viability. We isolated CD4+ T cells and CD8+ T cells from human peripheral blood mononuclear cells (PBMCs) using magnetic negative selection and transduced them with lentivirus encoding an anti-MSLN CAR featuring the SS1 anti-MSLN single-chain variable fragment (scFv) and the 41BBz signaling domain [43]. Transduction efficiency of CD4+ T cells and CD8+ T cells was determined based on the frequency of cells expressing the GFP reporter linked to CAR via a 2A peptide (Figure 6A). Anti-MSLN CAR CD4+ and CD8+ T cells were combined at a 1:1 ratio normalized for the number of CAR+ cells for in vitro cytotoxicity assays. Untransduced T cells were used as negative controls. To avoid potential negative effects of GEM on CAR T-cell cytotoxicity, we first treated cancer cells with GEM, then treated them with anti-MSLN CAR-T cells. Sequential targeting of SERPINB5 and MSLN with GEM and anti-MSLN CAR-T cells efficiently induced cancer cell death, more effectively than either agent alone (Figure 6B,C), as reflected in the reduced numbers of surviving cancer cells (Figure 6D,E). Overall, these findings indicate that sequential GEM and anti-MSLN CAR-T cell treatment is a promising therapeutic strategy for patients with pancreatic cancer, and that SERPINB5 levels may guide GEM dosing in this combination regimen.

## 4. Discussion

Pancreatic cancer is notoriously difficult to treat. Currently, surgery is the only potentially curative option for early-stage disease, yet fewer than 20% of patients are eligible. There is still no effective therapy capable of curing later-stage pancreatic cancer. A major recent advance is the KRAS-mutant-targeted drug daraxonrasib, which nearly doubled median overall survival from 6.7 to 13.2 months compared with chemotherapy in patients with metastatic pancreatic cancer [44]. Nonetheless, there remains an emergent unmet need to develop more effective therapeutic strategies for patients with pancreatic cancer.

This study demonstrates that SERPINB5 and MSLN are highly expressed in pancreatic cancer and that their expression levels are inversely correlated with overall survival in pancreatic cancer patients. Of note, this association is cancer-type-dependent (Figure 1C). Our analysis further revealed an inverse correlation between SERPINB5 and MSLN expression in pancreatic cancer cell lines. Genetic perturbation experiments showed that SERPINB5 downregulates MSLN expression. ChIP-qPCR data suggest that SERPINB5 expression suppresses c-Myc binding to the MSLN promoter, thereby decreasing MSLN expression. A follow-up promoter-reporter assay with a mutation could precisely identify the c-Myc-binding sites. Meanwhile, it is worth exploring whether SERPINB5 regulates c-Myc function directly or indirectly. Because nuclear SERPINB5 regulates gene transcription [45], future studies may also investigate whether and how it directly downregulates MSLN transcription.

Of note, both SERPINB5 and MSLN proteins are critical for pancreatic cancer survival, and simultaneous knockdown of SERPINB5 and MSLN significantly enhances apoptosis. Currently, no specific compound directly targets SERPINB5, whereas GEM remains a standard chemotherapy for pancreatic cancer. We found that high SERPINB5-expressing PC cells exhibited increased apoptosis in response to GEM, with significantly lower IC_50_ values than low-SERPINB5-expressing cells (Figure 4). In contrast, high-MSLN PC cells, which have low or undetectable SERPINB5 expression, showed a poor response to GEM. These findings suggest that PC sensitivity to GEM is SERPINB5-dependent, making SERPINB5 a useful biomarker for guiding GEM dosing in pancreatic cancer therapy. Our mechanistic studies indicate that SERPINB5 suppresses DHRS2 expression to prevent apoptosis. SERPINB5 knockdown or GEM treatment in high-SERPINB5-expressing pancreatic cancer cells increases DHRS2 levels, thereby inducing apoptosis. Notably, subsequent MSLN upregulation suppresses DHRS2 expression, leading to resistance to apoptosis (Figure 5). This mechanism may explain the clinical observation that pancreatic tumors often shrink initially with gemcitabine treatment but rapidly acquire resistance [46,47,48,49]. A follow-up study could include a time-course analysis and a model of acquired gemcitabine resistance. It is also worth testing whether preventing MSLN induction maintains DHRS2 expression and delays or reverses resistance. Although this study identifies SERPINB5-mediated downregulation of DHRS2 as a key pathway promoting cell survival, other SERPINB5-regulated mechanisms may also contribute to pancreatic cancer cell survival and warrant further investigation. DHRS2 upregulation may affect p53/CHKα/SPHK1 expression and mitochondrial functions [26,27,28,29]. It would be worth examining how SERPINB5 and MSLN regulate p53/CHKα/SPHK1 expression and mitochondrial function, and whether this regulation relies on DHRS2. Reactive oxygen species, mitochondrial membrane potential, ATP production, oxygen consumption rate, and cytochrome c release may be used to assess mitochondrial functions.

Anti-MSLN CAR T-cell therapy is an experimental immunotherapy for aggressive solid tumors that express high levels of MSLN on the surface, including malignant mesothelioma, pancreatic cancer, ovarian cancer, lung cancer, and triple-negative breast cancer. Clinical trials of anti-MSLN CAR-T cells have shown a high safety profile. The efficacy of anti-MSLN CAR T-cell therapy depends heavily on MSLN expression levels on tumor cells; low MSLN expression diminishes therapeutic success [50,51,52]. Our study showed that GEM treatment increases MSLN expression and that combining GEM with anti-MSLN CAR T-cell therapy induces greater cancer cell death. Although CAR-T cell therapy has limited efficacy in solid tumors, including pancreatic cancer, our study suggests that combining GEM with anti-MSLN CAR T-cells may enhance the clearance of blood-circulating pancreatic cancer cells and help prevent metastasis. Future studies may use orthotopic pancreatic cancer models to evaluate/confirm these in vitro findings and examine whether and how PC metastasis is regulated. In addition, GEM is a foundational chemotherapy drug for pancreatic cancer, and does not specifically regulate SERPINB5. Future work may focus on developing more specific strategies to target SERPINB5 for combination therapy with anti-MSLN CAR T-cells.

According to the Human Protein Atlas (proteinatlas.org), high-stage or metastatic PCs exhibit increased expression of MSLN and/or SERPINB5. Our study suggests that high-SERPINB5 PC cells display relatively low MSLN expression. PC tumors may therefore contain distinct subpopulations of cells, with some expressing high levels of SERPINB5 and others expressing high levels of MSLN. Pancreatic cancer cells with high SERPINB5 levels may initially respond to GEM treatment but subsequently develop resistance as MSLN expression increases. In parallel, heterogeneous PC cell populations with intrinsically high MSLN expression may also contribute to GEM resistance. Thus, simultaneously targeting both SERPINB5 and MSLN appears necessary for effective pancreatic cancer therapy.

This study has certain limitations. Figure 1 used a relatively small clinical sample set; it lacks in vivo validation of the combined effects of GEM and anti-MSLN CAR T cell therapy to assess tumor control, treatment sequence, toxicity, CAR-T-cell trafficking, and survival; and a mitochondria-associated mechanism is proposed but not yet functionally demonstrated.

## 5. Conclusions

Overall, this study identifies SERPINB5 and MSLN, both highly expressed in pancreatic cancer, as two critical proteins that promote cancer cell survival. Consequently, targeting both SERPINB5 and MSLN simultaneously represents a promising therapeutic strategy for pancreatic cancer.

## Data Availability

Data will be made available on request.

## Supplementary Materials

The following supporting information can be downloaded at https://www.mdpi.com/article/10.3390/cancers18193242/s1, Figure S1: Supplemental figures for Figure 2; Figure S2: Supplemental figures for Figure 3; Figure S3: Supplemental figures for Figure 4; Figure S4: Supplemental figures for Figure 5.
