# Supplementary material for: Targeting SERPINB5 and MSLN for Pancreatic Cancer Therapy
Source: Cancers (Basel). 2026 Oct 8;18(19):3242. doi: 10.3390/cancers18193242 (PMC13664176; doi:10.3390/cancers18193242)

Figure S1. Supplemental figures for Figure 2

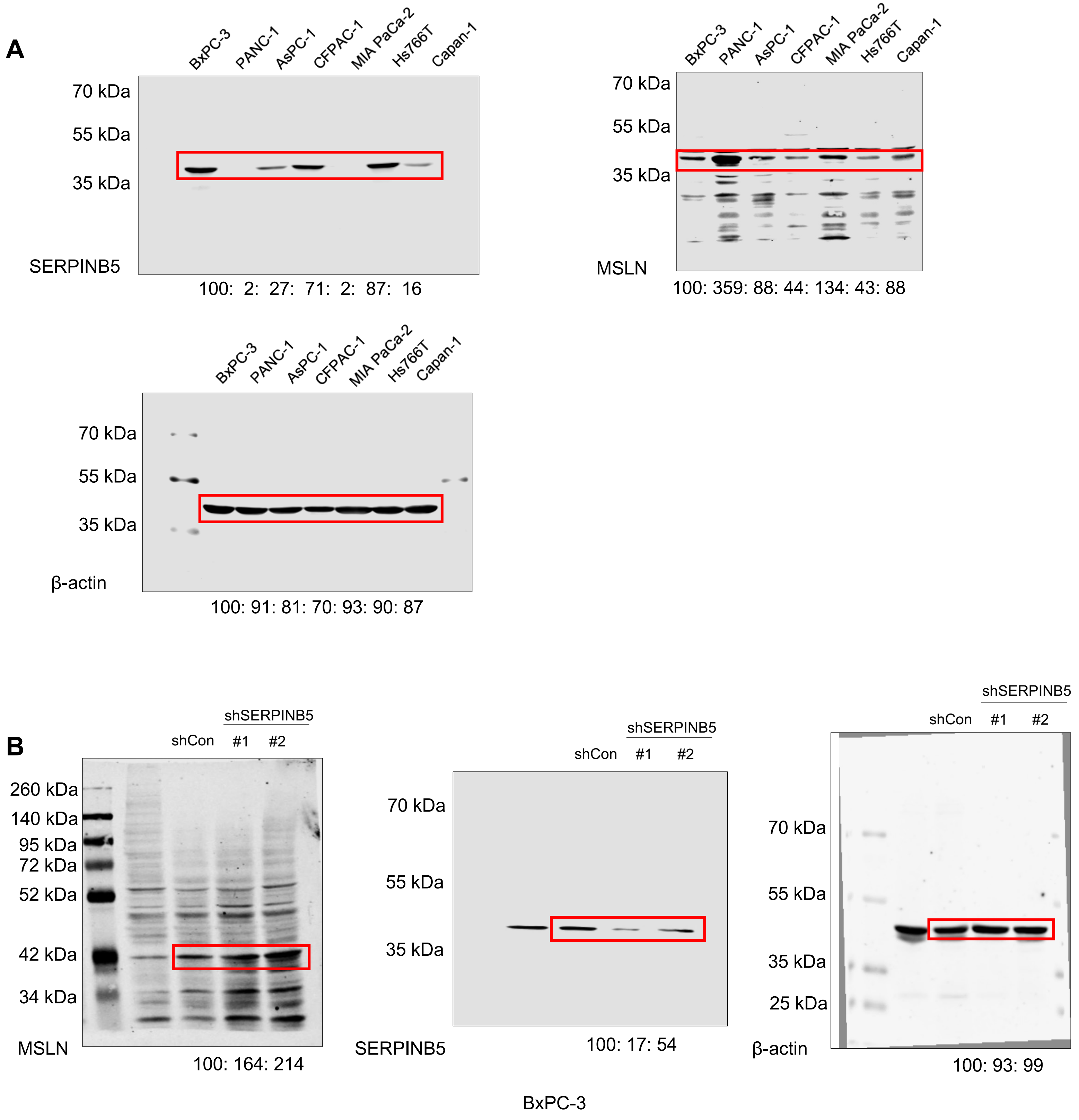

Figure S1. continued

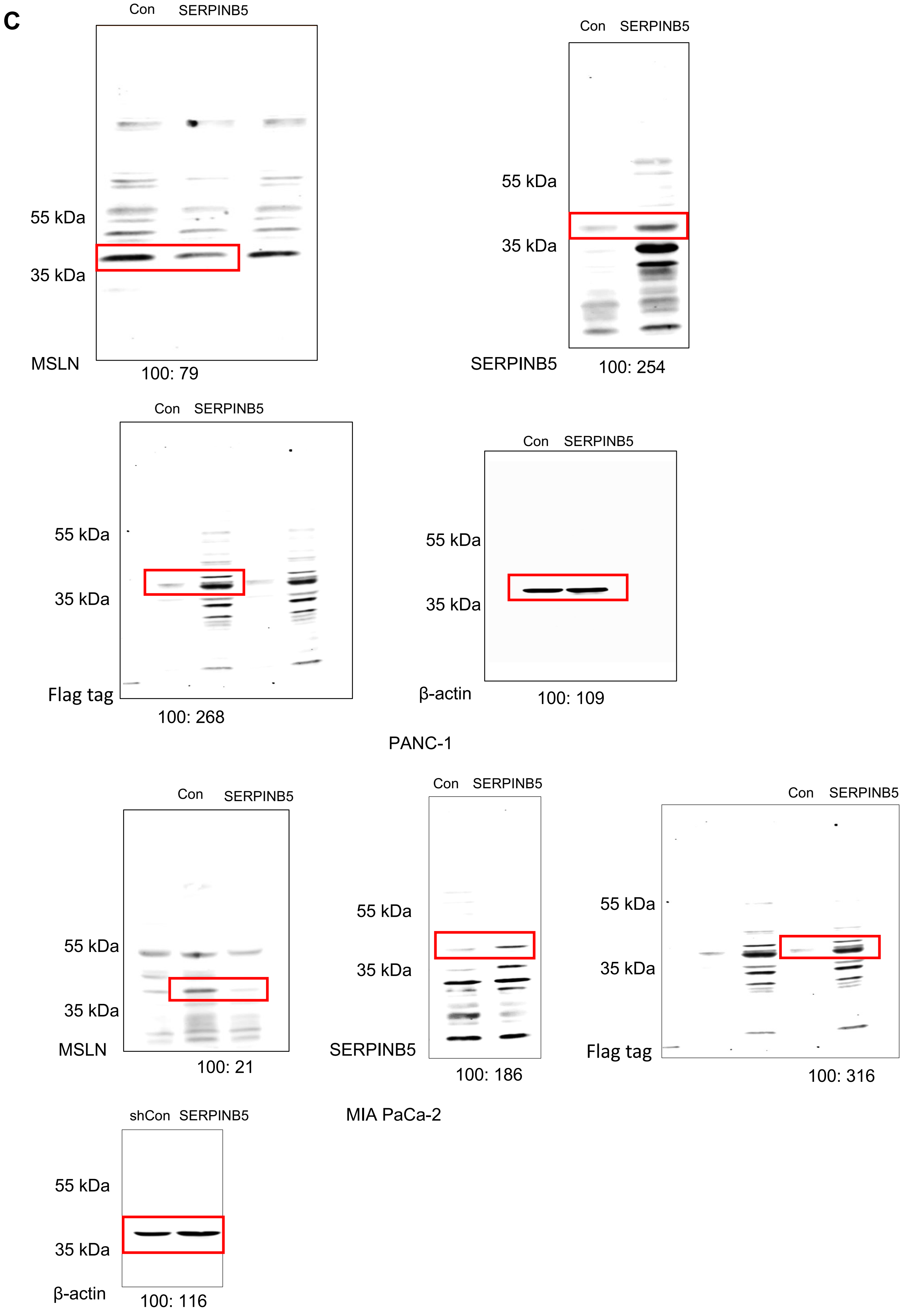

Figure S1. continued

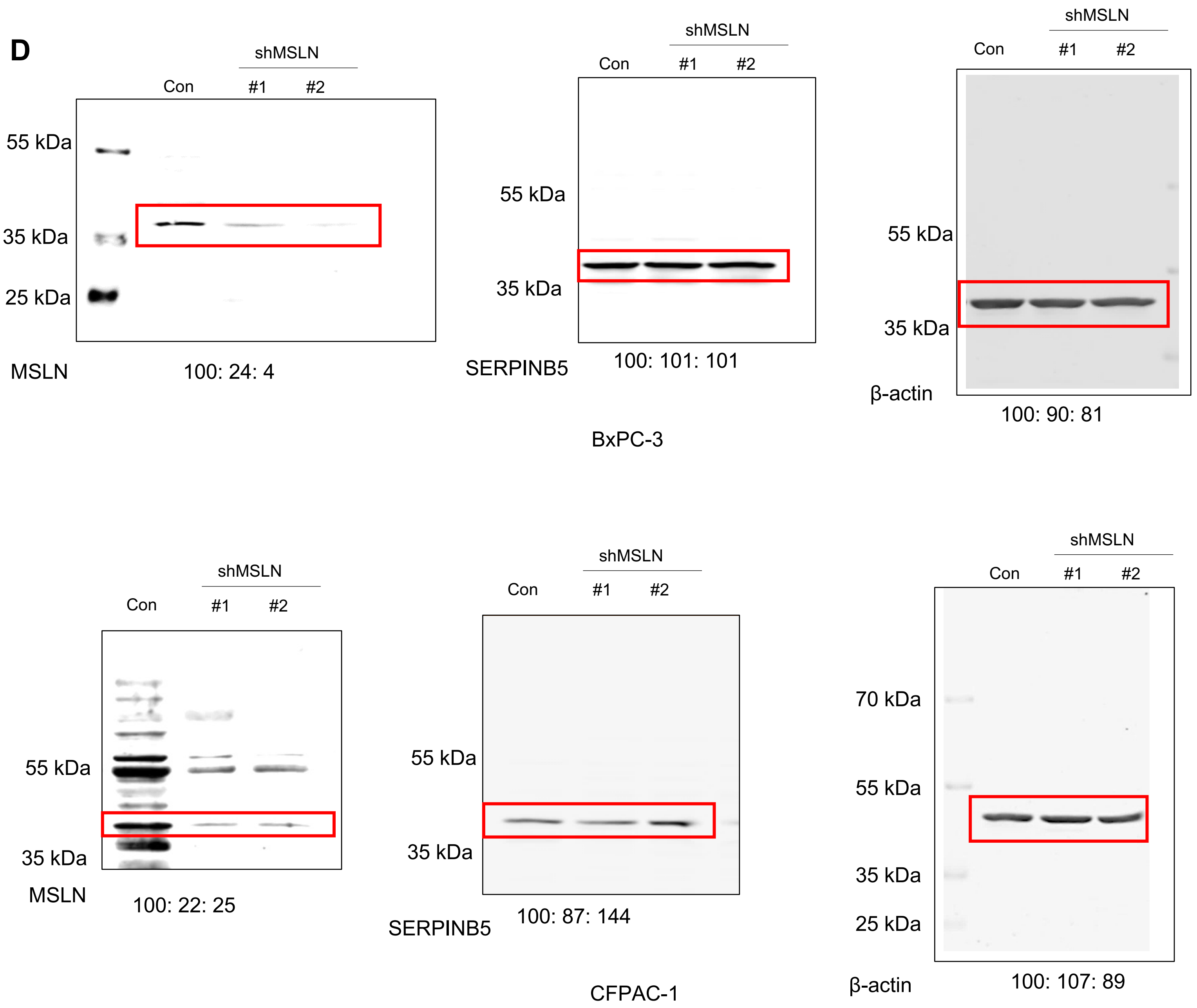

Figure S2. Supplemental figures for Figure 3

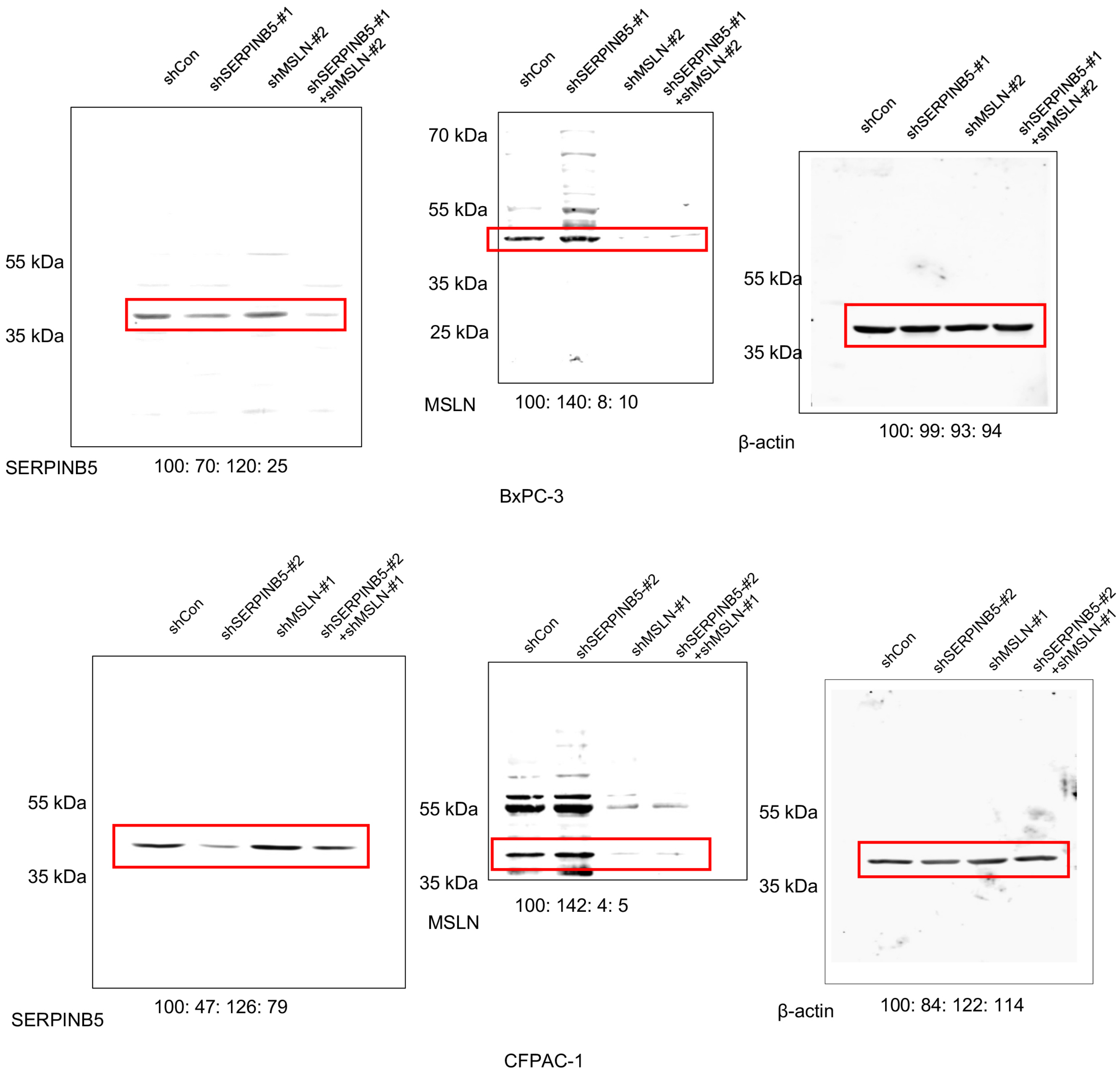

Figure S3. Supplemental figures for Figure 4

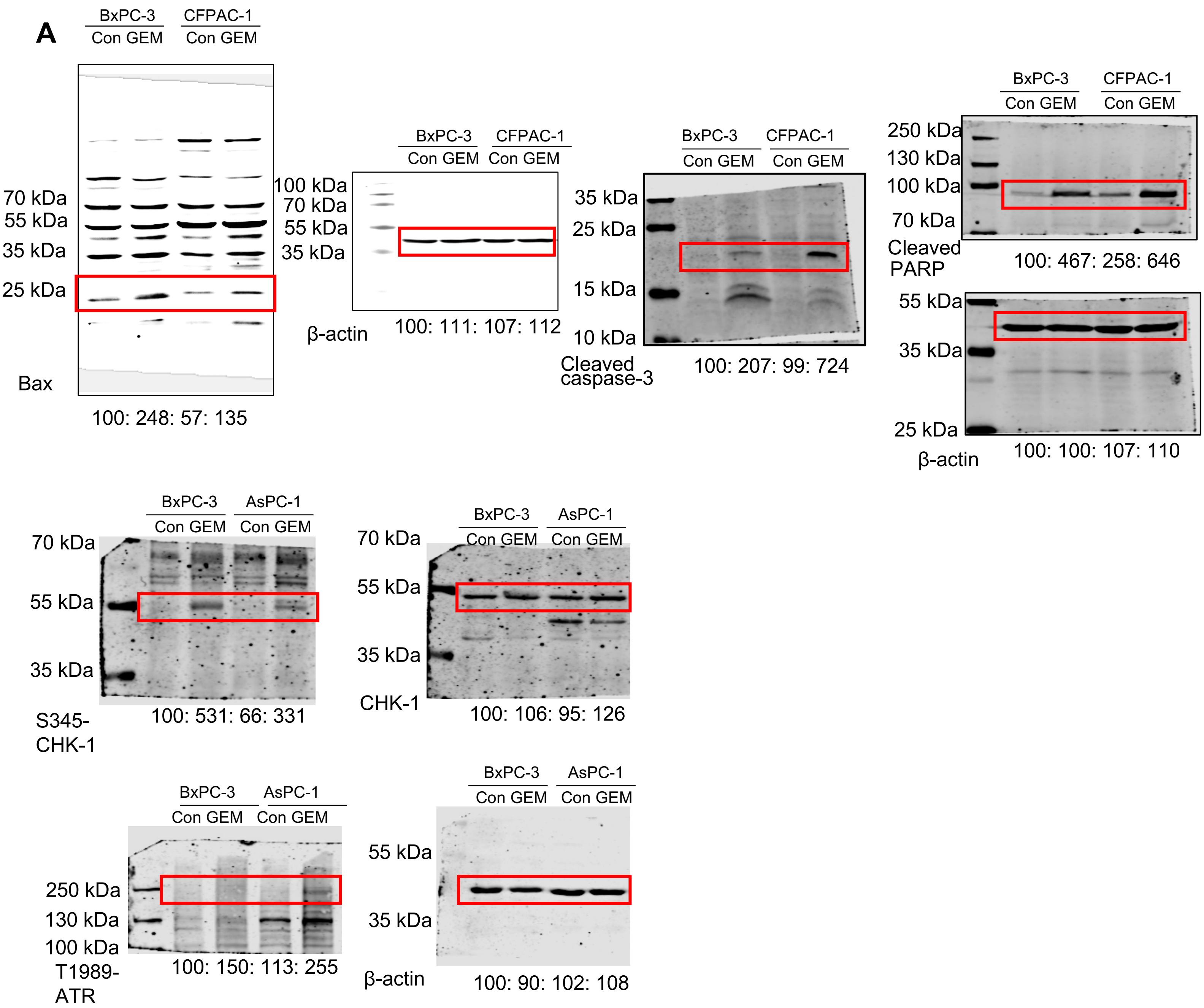

Figure S3. continued

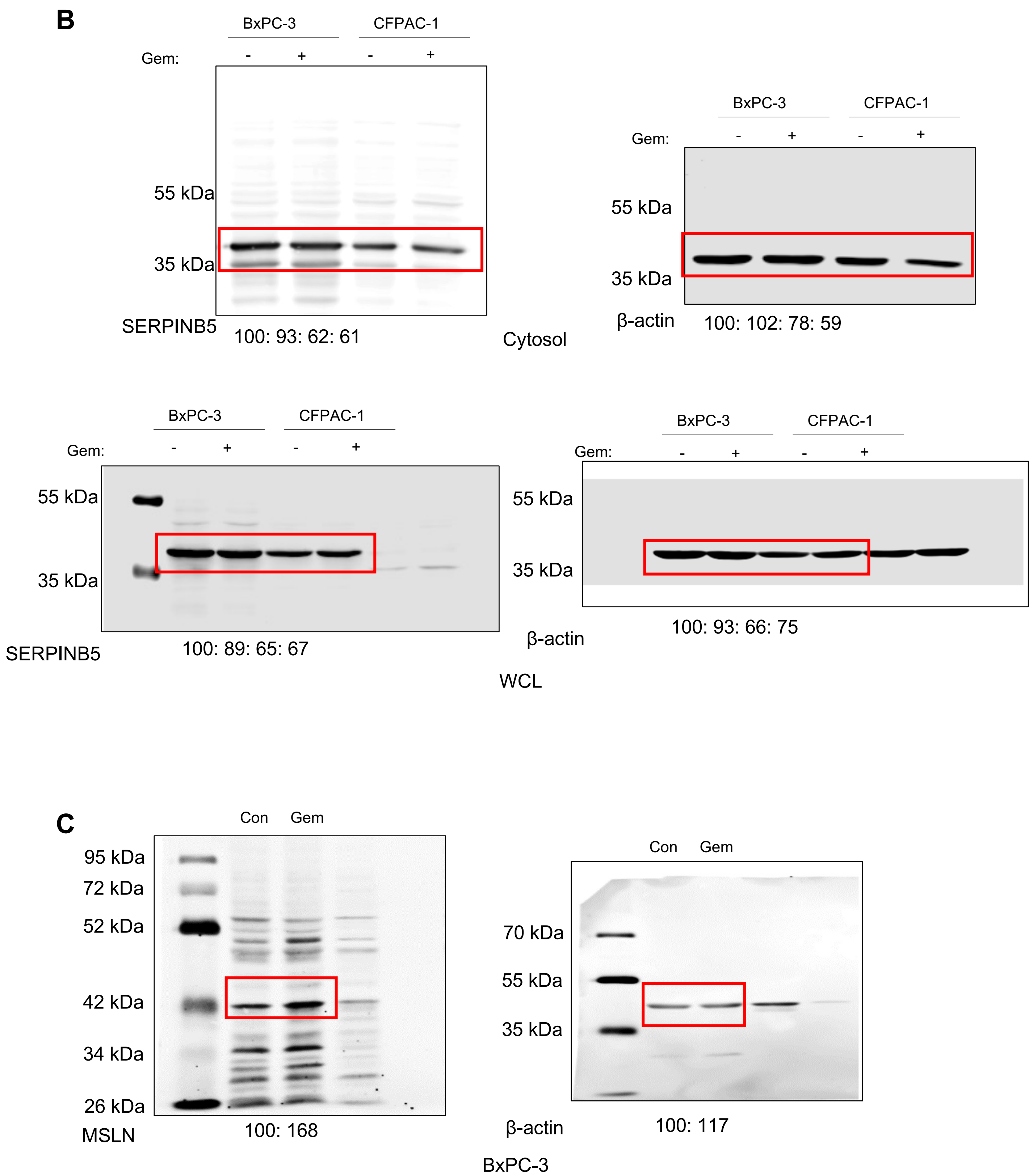

Figure S4. Supplemental figures for Figure 5

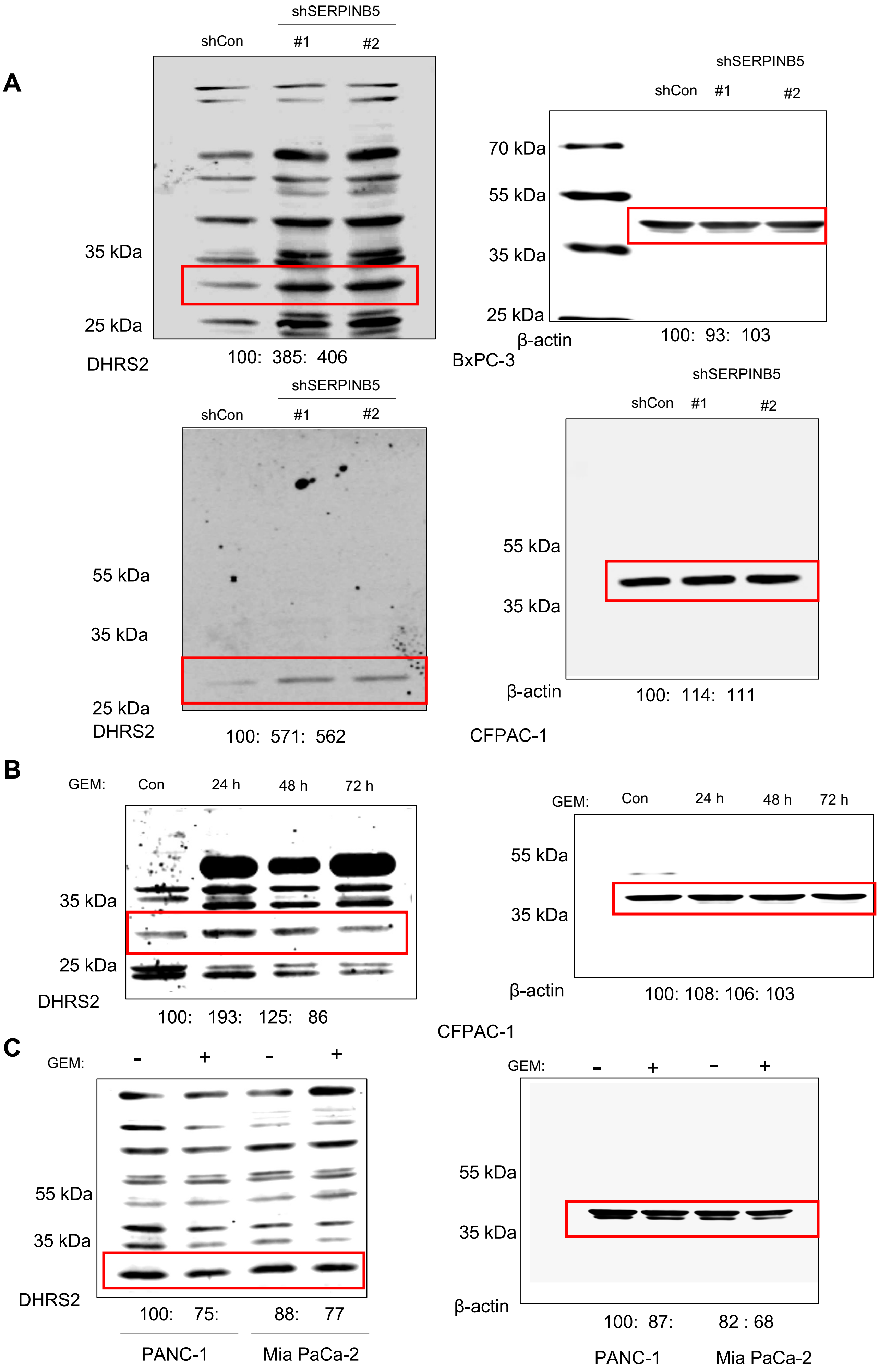

Figure S4. continued

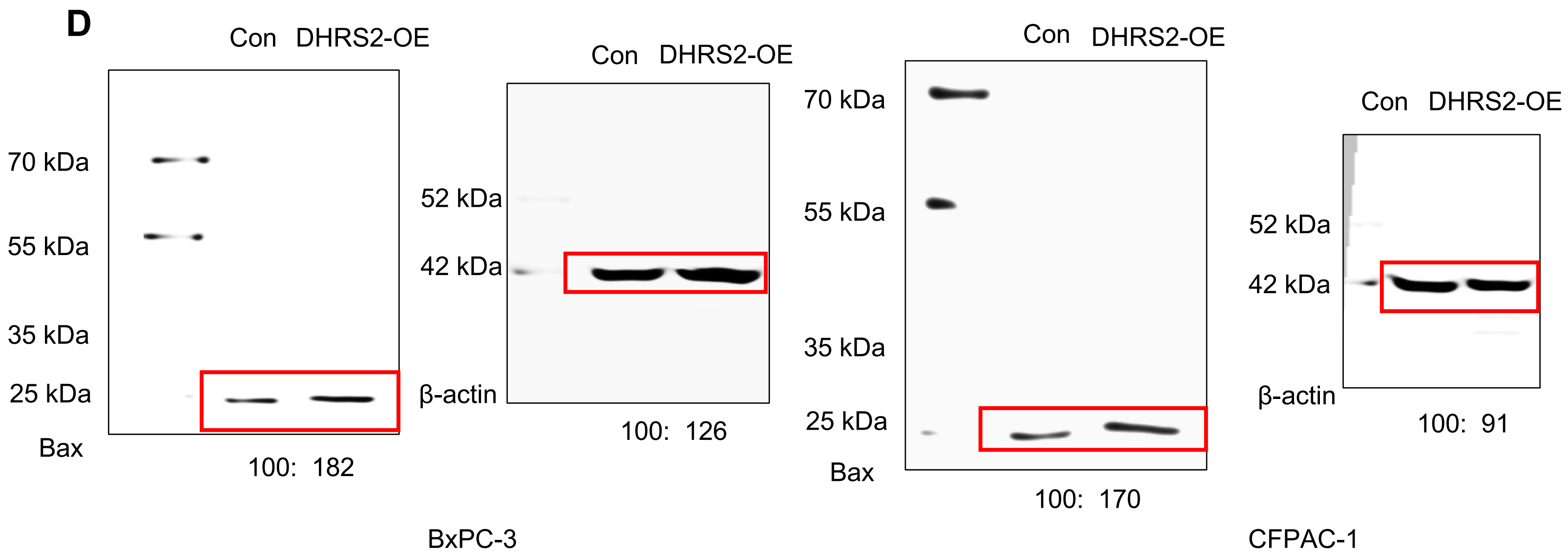

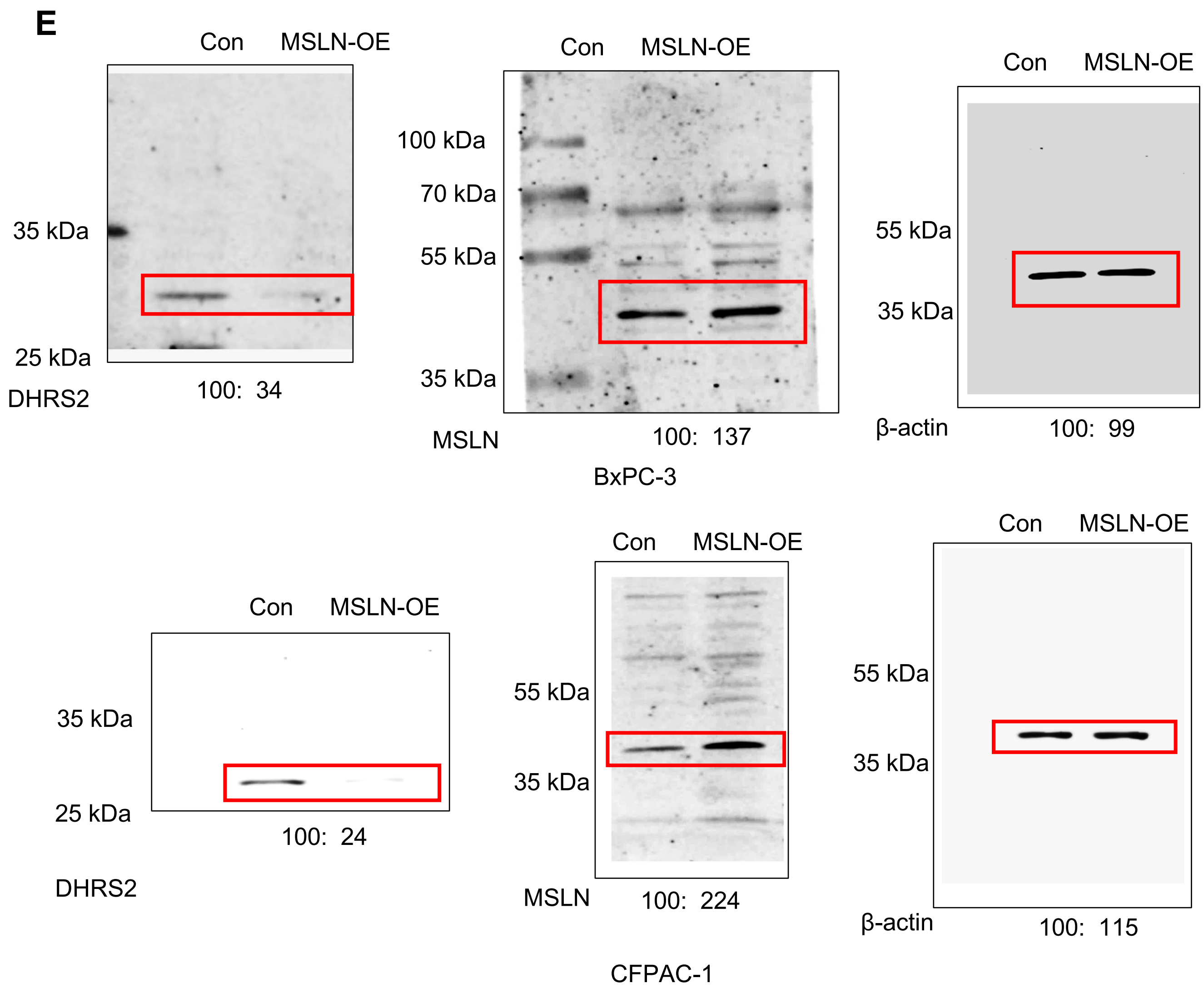

Figure S4. continued

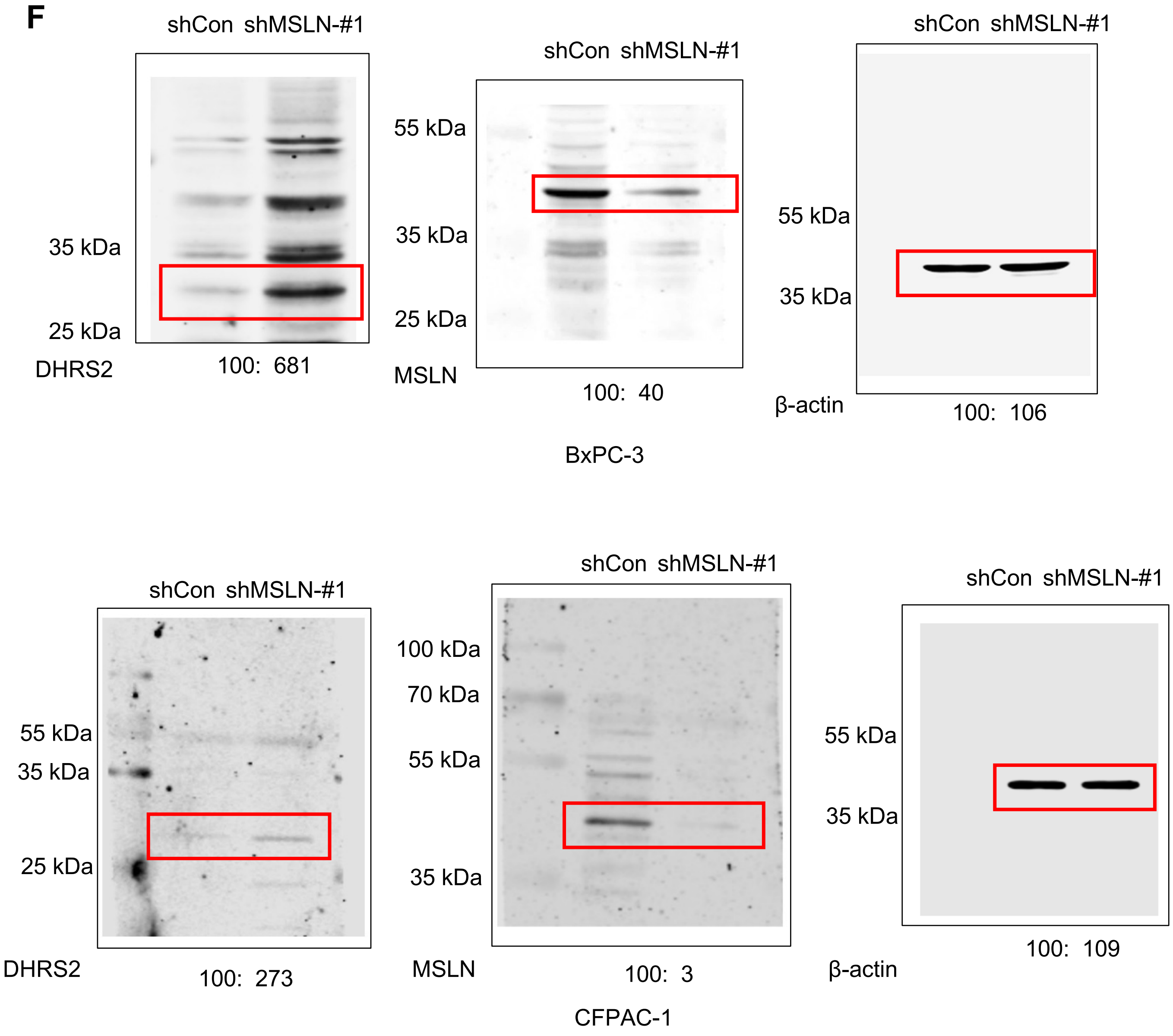

Supplement: Supplementary file 1 [file cancers-18-03242-s001.zip › cancers-4572040-supplementary.pdf]
